# Case Report: When genetic diagnosis comes late: lessons from a DEND syndrome patient successfully transitioned to sulfonylurea

**DOI:** 10.3389/fcdhc.2025.1654037

**Published:** 2025-10-15

**Authors:** Gabriella de Medeiros Abreu, Ritiele Bastos de Souza, Marília Chaves Bernardo, Jardeson Lima da Cruz Junior, Amanda Ferreira, Deborah Snaider, Ana Carolina Proença da Fonseca, Kaio Cezar Rodrigues Salum, Verônica Marques Zembrzuski, Marco Antonio Lima, Roberta Magalhães Tarantino, Melanie Rodacki, Lenita Zajdenverg, Jorge Luiz Luescher, Eliane Lopes Rosado, Mário Campos Junior

**Affiliations:** ^1^ Instituto de Nutrição Josué de Castro, Universidade Federal do Rio de Janeiro, Rio de Janeiro, Brazil; ^2^ Laboratório de Genética Humana, Instituto Oswaldo Cruz, Fundação Oswaldo Cruz, Rio de Janeiro, Brazil; ^3^ Departamento de Diabetes e Nutrologia, Hospital Universitário Clementino Fraga Filho, Universidade Federal do Rio de Janeiro, Rio de Janeiro, Brazil; ^4^ Laboratório de Imunofarmacologia, Instituto Oswaldo Cruz, Fundação Oswaldo Cruz, Rio de Janeiro, Brazil; ^5^ Laboratório de Genética, Universidade do Grande Rio/AFYA, Rio de Janeiro, Brazil; ^6^ Programa de Pós-graduação em Biomedicina Translacional, Universidade do Grande Rio/AFYA, Rio de Janeiro, Brazil; ^7^ Laboratório de Pesquisa Clínica em Neuroinfecções, Instituto Nacional de Infectologia Evandro Chagas, Fundação Oswaldo Cruz, Rio de Janeiro, Brazil; ^8^ Instituto de Puericultura e Pediatria Martagão Gesteira, Universidade Federal do Rio de Janeiro, Rio de Janeiro, Brazil

**Keywords:** neonatal diabetes mellitus, DEND syndrome, *KCNJ11*, Kir6.2, sulfonylurea, late diagnosis

## Abstract

Neonatal diabetes mellitus (NDM) is a rare cause of diabetes characterized by the presence of severe hyperglycemia typically diagnosed within the first six months of life. Among the main causes are activating variants in heterozygosity in the *KCNJ11* gene. Variants in this gene can lead to a spectrum of clinical manifestations, from transitory neonatal diabetes mellitus to DEND syndrome, the most severe form, characterized by developmental delay, epilepsy, neonatal diabetes, and muscle hypotonia. The disease may be present in a milder intermediate form named iDEND syndrome. Patients with *KCNJ11* variants may present with attention deficit hyperactivity disorder (ADHD), autism spectrum disorder (ASD), developmental coordination disorder (DCD), and learning difficulties due to diminished intelligence quotient (IQ) and dyslexia. These patients can benefit from genetic counseling as most of them can switch from insulin to sulfonylurea treatment with good glycemic control and no severe side effects; besides, some studies report a neurological improvement after the treatment switch. In the present work, we reported a follow-up of a 24-year-old Brazilian male with DEND syndrome due to the *KCNJ11* c.754G>A; p.(Val252Met) variant. He was diagnosed with diabetes at 25 days of age and presented with bilateral hypoacusis in the first years of life. He started insulin at the diagnosis. However, the genetic diagnosis was made only at the age of 15 years, and he was switched from insulin to sulfonylurea. At 24 years of age, he presents with good glycemic control and reports no severe episodes of hypoglycemia or hyperglycemia. However, no neurological improvement was observed. This report highlights the potential benefits of switching to sulfonylurea treatment, even in patients with long-standing diagnoses of DEND syndrome, and underscores the importance of genetic diagnosis, as early initiation of sulfonylurea therapy may improve metabolic control and, in some cases, neurological outcomes.

## Introduction

Neonatal diabetes mellitus (NDM) is characterized by the presence of severe hyperglycemia typically within six months of life and less commonly between six months and one year (1). NDM occurs generally due to a variant in a single gene that affects the pancreatic beta cell function and consequently affects the level of circulating insulin (2, 3). Its incidence varies widely from 1/89,000 to 1/260,000 among different ethnic cohorts (4–8).

There are two distinct forms of NDM based on the duration of pharmacological treatment; in the transient NDM form (TNDM) hyperglycemia usually resolves at a young age, although it has a high recurrence rate in adolescence, while patients with permanent NDM (PNDM) form require life-long treatment (1, 2).

Since the observation of the first genetic causes of NDM, the number of causal genes is still increasing and more than 40 genes have been described over the years (9). However, three genetic alterations are more frequent in NDM cases: abnormalities of the 6q24 locus methylation, and activating variants in the *ABCC8* and *KCNJ11* genes, which encode the SUR1 and Kir6.2 subunits, respectively, of the ATP-dependent potassium channel (KATP channel) (10).

In response to a rise in glucose blood level, the KATP channel closes, leading to insulin secretion from the pancreatic β-cells into the bloodstream. Activating variants in the *ABCC8* and *KCNJ11* impair the KATP channel closure, preventing the cascade that induces insulin secretion (10). The majority of heterozygous activating variants in *KCNJ11* appear to occur *de novo* and are responsible for most cases of NDM in non-consanguineous cohorts, with over half of these cases presenting with central nervous system (CNS) involvement (1, 2, 11–14). The KATP channels are also expressed in neurons, and severe variants in *KCNJ11* may increase these channels’ activities (15).

Some patients with *KCNJ11* activating variants can present a spectrum of neurological abnormalities. Severe forms of this condition can present developmental delay, epilepsy, NDM, and muscle hypotonia (DEND syndrome), or iDEND syndrome, a mild form in which epilepsy is absent (13, 16). Additionally, patients with activating variants in *KCNJ11* may present with attention deficit hyperactivity disorder (ADHD), autism spectrum disorder (ASD), anxiety disorders, sleep difficulties, developmental coordination disorder (DCD), delays in learning, diminished intelligence quotient (IQ), and dyslexia (1, 14, 17–19).

More than 90% of patients with NDM caused by *KCNJ11* variants benefit from sulfonylureas (SU) treatment reporting good glycemic control (glycated hemoglobin (HbA1c): ~5.9-6.2%) and with no severe hypoglycemic episodes reported (14, 20–24). SU acts by binding within the transmembrane domain of the SUR1 subunit, promoting the block of the KATP channel, and stimulating the endocrine insulin release by pancreatic β-cells (25). The use of SU has been proven safe and effective for at least 10 years of treatment and shows fewer risks of hypoglycemia than insulin injections (14, 26).

The location and the type of variant seem to be linked to the phenotype and response to SU. This may explain why some patients are unable to transition to SU and continue on insulin treatment (15, 24). Also, it seems that an increase in age at initiation of SU results in an increased dose required (23). This phenomenon may be due to prolonged exposure to hyperglycemia, which seems to result in a decrease in β-cell mass (27). Besides, microvascular complications in patients with *KCNJ11* variants are improbable but were observed in those patients transferred to SU from insulin later (median age: 20.5 years) (14). Higher SU doses have been reported (0.84-2.4 mg/kg) to improve neurological and psychomotor abnormalities (1, 14, 28–30), with a better response if it is initiated at an early age (30).

The early genetic diagnosis of patients with NDM allows better glycemic control, prognosis, and quality of life (26). In the present work, we report a follow-up of a 24-year-old Brazilian patient with DEND syndrome who received the genetic diagnosis 15 years after the onset of diabetes.

## Case description

The proband is a Brazilian male, third child of non-consanguineous parents. At the time of his birth, the mother was 25 years old and the father was 26. The patient was born at term with a birth weight of 3,000 g and a length of 49 cm from spontaneous vaginal delivery and was discharged home three days after birth. He was exclusively breastfeeding in the first month of his life.

At 25 days of life, the proband developed acute gastroenteritis with severe dehydration that progressed to sepsis, respiratory failure, and cardiopulmonary arrest. He went on mechanical ventilation for seven days. During the hospitalization, he presented diabetic ketoacidosis with hyperglycemia, hypernatremia, and metabolic acidosis. He was diagnosed with DM and received continuous insulin infusion for three days. After that, the treatment was transitioned to subcutaneous insulin therapy, using 2 units of NPH insulin and regular insulin, according to self-blood glucose monitoring (SBGM).

At two months, the capillary glucose measurement ranges were as follows: at 6:00 am, 200–300 mg/dL; at 12:00 pm, 40–80 mg/dL; at 6:00 pm, 80–200 mg/dL; and at 12:00 am, 120–300 mg/dL. At this time, right convergent strabismus was observed, and, in the fifth month, he developed severe bilateral hypoacusis. At the age of one, the HbA1c was 7.4%.

At the age of seven years (HbA1c: 6.1%), the assessment of orofacial muscle tone evaluation revealed hypotonic cheeks with mouth breathing and dyslalia.

At nine years (HbA1c 5.5%), the patient experienced two episodes of loss of consciousness with bilateral limb flexion, without evidence of hypoglycemia; at this time, he was at 0.16 U/kg/day of NPH insulin. The electroencephalogram (EEG) revealed paroxysmal events mainly in the temporal lobe. The neurological evaluation revealed incoordination, a sporadic slight tremor in the hands, mild diffuse spasticity in all, and atypical gait. He was initiated on phenobarbital (3 mg/kg/day), but after two months, the patient had two seizures; therefore, the phenobarbital dose was increased to 4 mg/kg/day. After this, he had another episode at the age of ten years (HbA1c: 6%). At this age, the patient remained illiterate and could not recognize colors. The EEG at 12 years of age (HbA1c: 5.7%) revealed focal slow and sharp waves.

At the age of 15 years, he received the genetic diagnosis of NDM with a variant in the *KCNJ11* gene. After the genetic diagnosis, the patient started treatment with glyburide 0.2 mg/kg/day; Insulin doses were gradually tapered while glyburide therapy was initiated and titrated in parallel. Dose adjustments for both insulin and glyburide were guided by capillary blood glucose profiles obtained by the patient, with close monitoring to maintain glycemic stability until complete discontinuation of insulin was achieved. However, between the ages of 15 and 20, the patient showed poor treatment adherence, frequently discontinuing medication without medical guidance. His highest recorded HbA1c during this period was 17.2%. At age 16, he independently stopped phenobarbital, after which no further seizures occurred.

A few months after starting glyburide (0.2 mg/kg/day), he experienced hypoglycemic symptoms (sweating, tremors) and discontinued the medication. At 17, he resumed treatment for six months, with fasting glucose of 137 mg/dL, HbA1c of 7.2%, and capillary glucose between 130–149 mg/dL. He again stopped treatment for three months, reporting polyuria and capillary glucose between 150–200 mg/dL.

Treatment was restarted for another six months, with a maximum capillary glucose of 139 mg/dL and one self-reported hypoglycemic episode. He discontinued treatment once more. At age 19, his HbA1c rose from 12.4% to 17.2%. Subsequently, the glyburide dose was reduced to 0.1 mg/kg/day, eliminating hypoglycemic symptoms and improving adherence. From ages 20 to 24, HbA1c remained between 5.4% and 7.4%.

**Figure 1:**
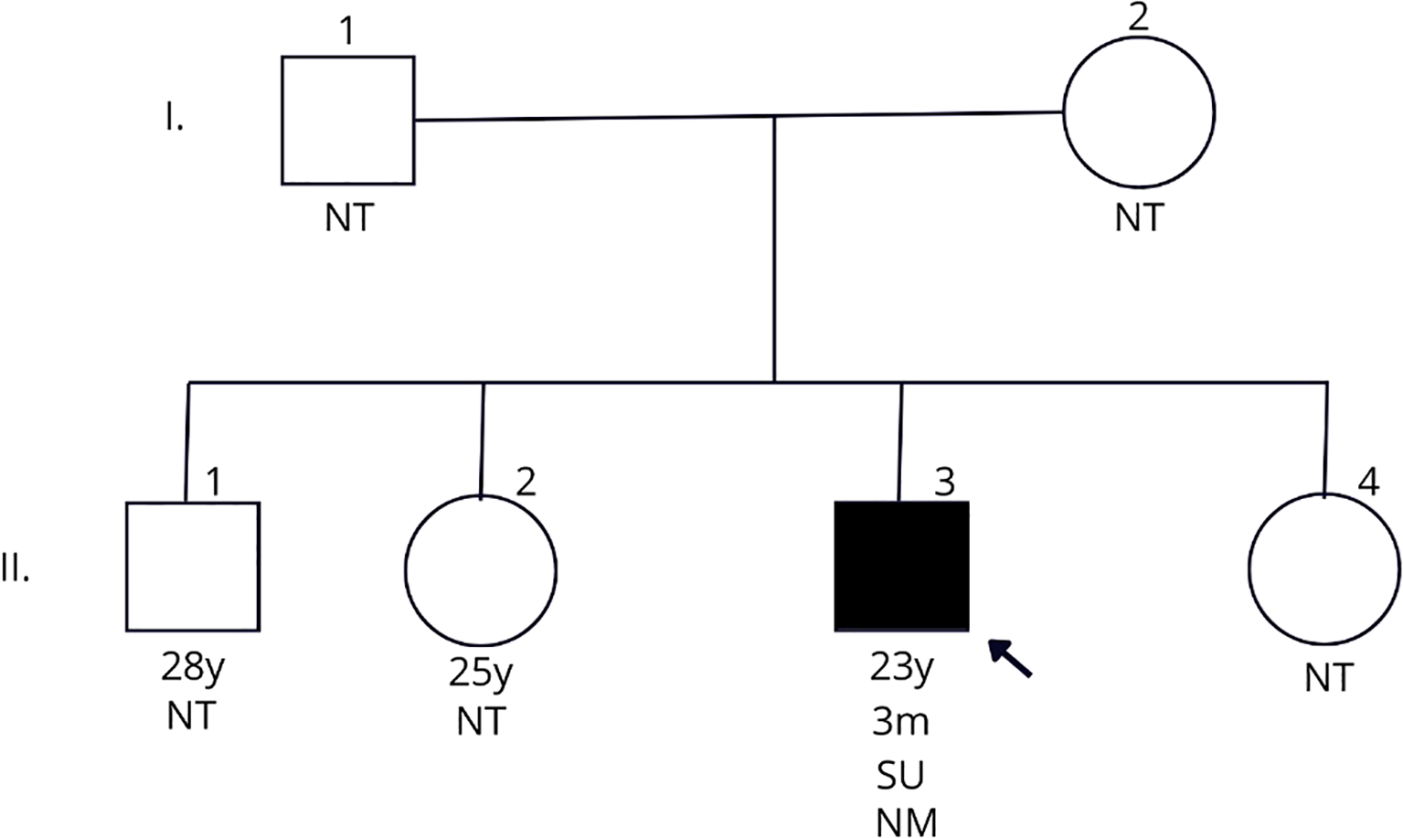
Follow-up of HbA1c (orange line) variation according to the treatment by 4 years to 24 years old of the patient carrier of *KCNJ11* c.754G>A variant. Until the age of 13 years, the proband was treated with 4 U of insulin NPH (grey bar). Sulfonylurea (yellow bar) was started at 15 years old. At 14 and 19 years the patient refused to receive the treatment.

**Figure 2:**
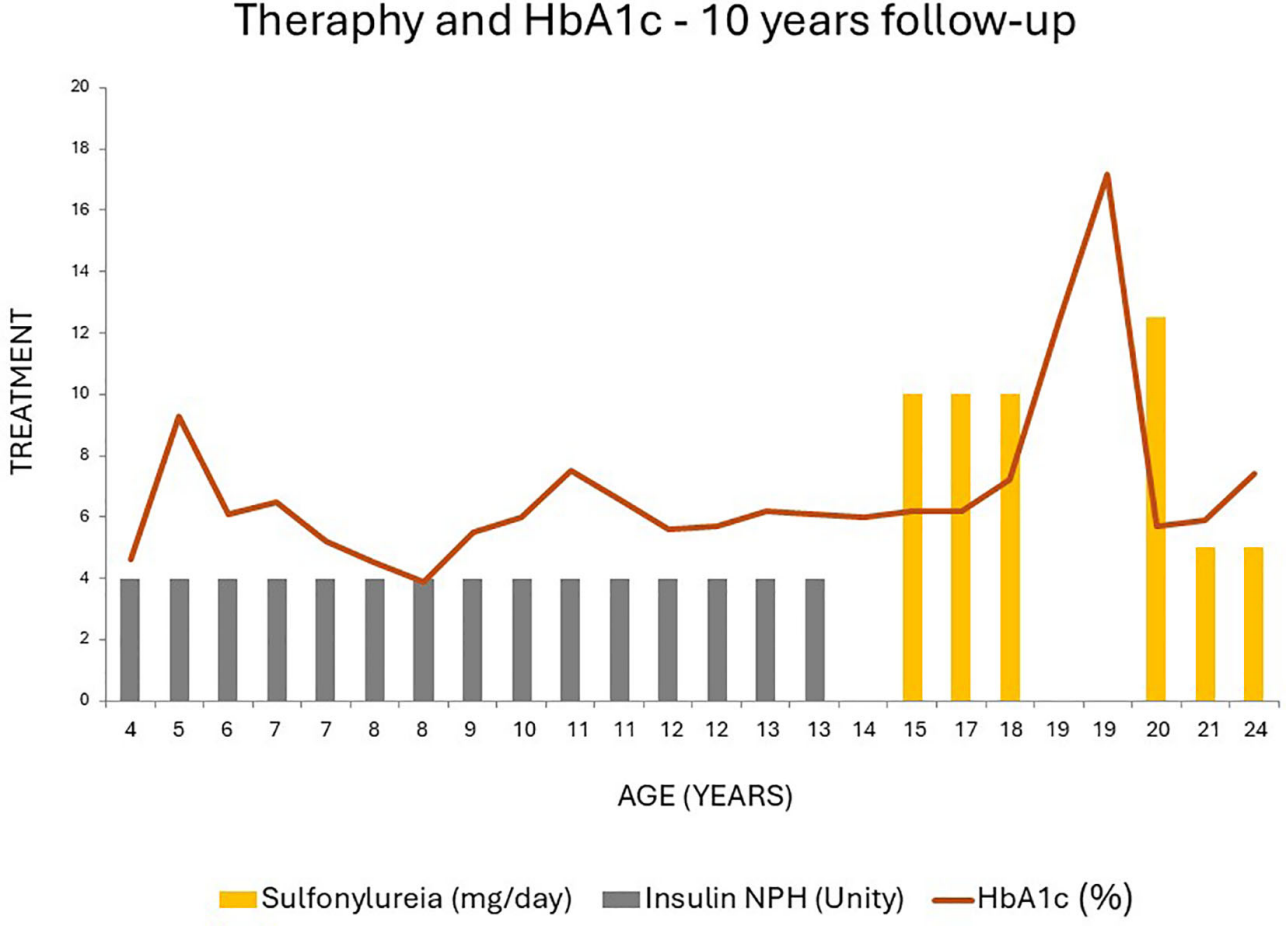
Pedigree of the family. Filled symbols represent DEND syndrome and empty symbols non self-reported diabetic individuals. The present age of the individuals is shown below the symbols, followed by age of diagnosis, the most recent treatment, body mass index (kg/m2), and genotype interpretation. SU: Sulfonylurea treatment; Genotypes of KCNJ11 c.754G>A are expressed by normal allele (N) and mutated allele (M); NT, Not tested; y, years; m, months. An arrow indicates the index case.

During follow-up at Hospital Clementino Fraga Filho, the patient maintained normal weight, showed detectable C-peptide after 15 years of disease (1.1 ng/mL), and had no diabetes-related complications. Acanthosis nigricans and hypertriglyceridemia were not observed. HbA1c trends are illustrated in **Figure 1**, and the pedigree in **Figure 2**. Family members reported no glycemic or neurological symptoms and were unavailable for genetic testing.

## Diagnostic assessment

### Genetic analysis

Genomic DNA from the proband was isolated from peripheral blood leukocytes using the QIAamp DNA Blood Mini Kit (Qiagen, Hilden, Germany). The entire coding region of the *KCNJ11* gene was screened by Sanger sequencing. Polymerase chain reaction (PCR) was carried out by amplification of three overlapping fragments using three pairs of primers (KCNJ11_A Forward 5’ AGAGTCTGGTGGGGAGTTATCT 3’ and KCNJ11_A Reverse 5’ GGGCACTCCTCAGTCACC 3’; KCNJ11_B Forward 5’ TCTTCACCATGTCCTTCCTGTG 3’ and Reverse 5’ TCGTAGAGTGGGCTGTTGG 3’; KCNJ11_C Forward 5’ TGGCCCCGCTGATCATCTA 3’ and KCNJ11_C Reverse 5’ GCCGGGCTACATACCACAT 3’). PCR products were purified by ExoSAP-IT™ PCR Product Cleanup Reagent (Applied Biosystems, Vilnius, Lithuania) and bidirectional Sanger sequencing was performed using the Big Dye Terminator Kit v3.1 (Applied Biosystems, Austin, TX, USA), conducted on an ABI 3130 Automatic Genetic Analyzer (Applied Biosystems). The variant identified in the proband was confirmed by bidirectional sequencing of a second PCR reaction.

### Bioinformatic analysis

The variant identified was checked against public Databases: gnomAD - Genome Aggregation Database (v2.1.1) (https://gnomad.broadinstitute.org/), ABraOM - Online Archive of Brazilian Mutations Online (https://abraom.ib.usp.br/), dbSNP - Database of single nucleotide polymorphisms (https://www.ncbi.nlm.nih.gov/snp/), HGMD (https://www.hgmd.cf.ac.uk/ac/) and ClinVar (https://www.ncbi.nlm.nih.gov/clinvar/). The variant was classified following the guidelines of the ACMG/AMP - American College of Medical Genetics and Genomics and the Association for Molecular Pathology (31) available on Franklin (https://franklin.genoox.com/clinical-db/home). The location of the variant identified was visualized by Pymol molecular graphics system using the Protein Data Bank (PDB) (https://www.rcsb.org) under the ID: 6BAA (32).

**Figure 3:**
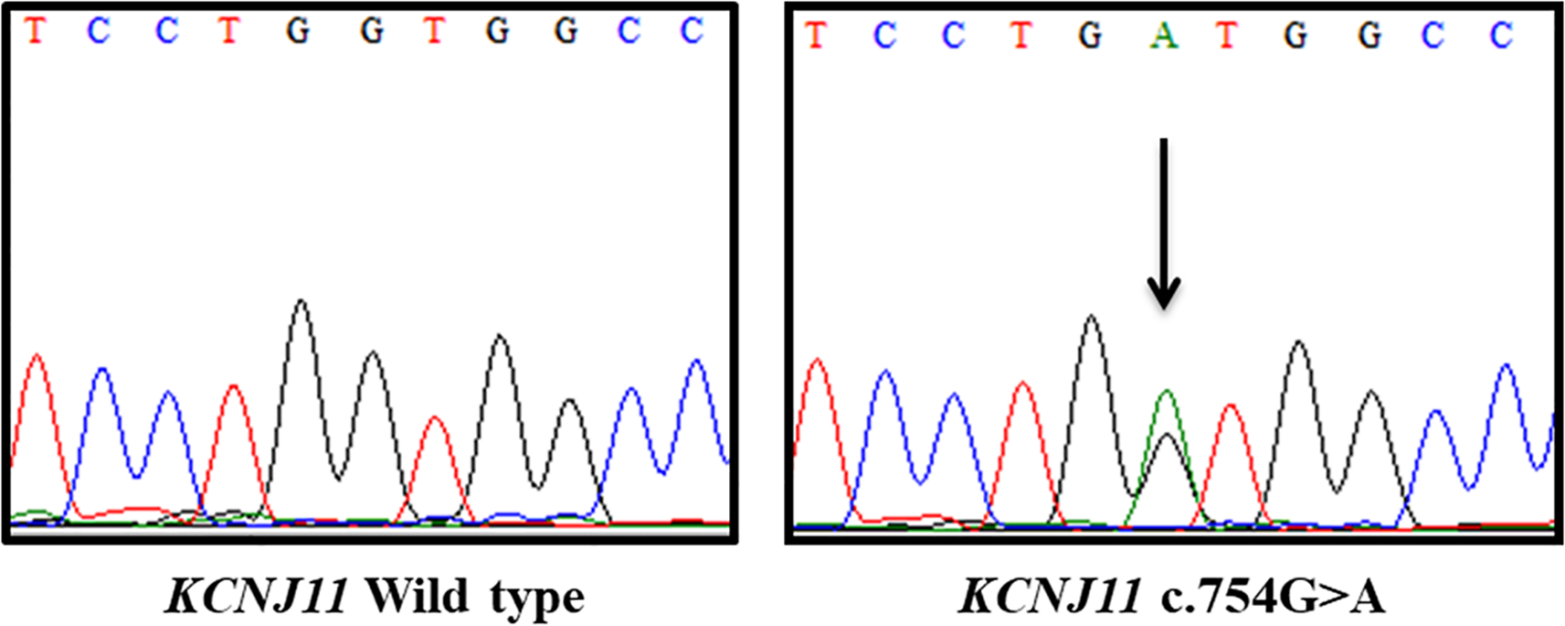
Electropherograms of *KCNJ11* wild type (left) and *KCNJ11* c.754G>A in heterozygosis identified in the proband (right).

**Figure 4:**
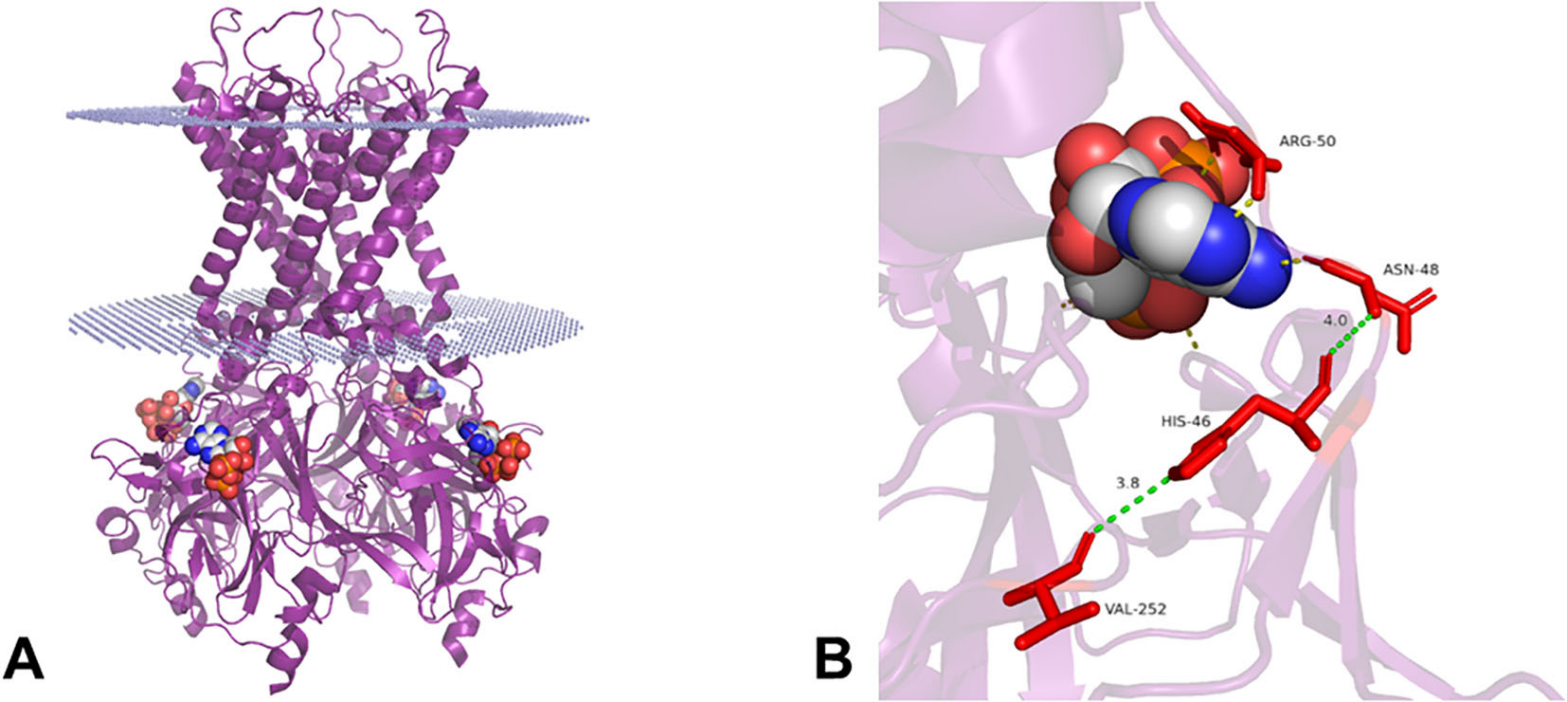
3D structure of Kir6.2 tetramer (purple) retrieved from the Protein Data Bank (PDB) under the ID: 6BAA. The image was generated using the Pymol molecular graphics system. The dotted structure (blue) shows the positioning approximate of protein in the membrane. ATP is shown as spheres colored by atom carbon (white), nitrogen (blue), oxygen (red), and phosphorous (orange) **(A)**. The binding site shows Kir6.2 residues that interact with ATP (yellow lines) through polar contacts. Below, position 252 (red) shows the location of the residue with alteration found in this study and the distance in angstroms **(Å)** (green lines) between the residues VAL252, HIS46, and ASN48 (red) **(B)**.

The screening of the *KCNJ11* reveals a c.754G>A;p.(Val252Met) variant in the heterozygous state (**Figure 3**). This variant was not found in the public databases accessed. It was classified as Likely pathogenic by ACMG guidelines (PM5, PP3, PM2, PM1 and, PP2 criteria). The variant location on the Kir6.2 protein is shown in **Figure 4**.

## Discussion

In this study, we reported a case of a male diagnosed with DEND syndrome, a very rare disorder with an incidence of less than 1 in 1,000,000 (33). Additionally, besides its rarity, its clinical diagnosis is even more challenging due to its pleiotropic manifestation, impacting the treatment choice. Our patient received the diagnosis of DEND syndrome at the age of 15 when he started SU treatment.

According to the International Society for Pediatric and Adolescent Diabetes (ISPAD) (2022) and the Brazilian Diabetes Society (2020), all infants diagnosed in the first six months of life should be screened for neonatal diabetes genes (34, 35). Genetic screening allows the diagnosis of monogenic diabetes in more than 80% of cases (2). Nonetheless, in countries where genetic testing for neonatal diabetes is not covered by the public healthcare system—such as Brazil—and remains financially inaccessible through private laboratories, its implementation is significantly constrained. Beyond the inherent rarity of the condition, this lack of access further hampers scientific dissemination and contributes to frequent misdiagnoses by clinicians unfamiliar with the disease. Additionally, treatment adherence may be influenced by limited disease understanding, often associated with low educational attainment and socioeconomic vulnerability, as observed in the present case.

Previous studies also highlight the importance of early genetic diagnosis in optimizing treatment. In cases where early genetic diagnosis is not feasible, the decision to switch the insulin treatment to SU should be weighed before considering an empiric trial (36). Besides, it should be taken into consideration even in cases of delayed diagnostic.

Vendramini and colleagues (2010) reported a case of a patient genetically diagnosed with iDEND syndrome at the age of 26 years and demonstrating good glycemic response to the transfer from insulin to SU; this data shows the importance of the correct diagnosis even during adulthood (37). Mancioppi and colleagues (2023) described a case of a patient with PNDM that, despite the late diagnosis at age 18, the patient’s treatment was switched from insulin therapy to glibenclamide at age 5, resulting in favorable glycemic outcomes and improvements in language development (38).

In the majority of NDM cases caused by *KCNJ11* variants, diabetes will appear within the 3 months of life (13). At the time of presentation, infants with PNDM may experience dehydration and diabetic ketoacidosis (DKA) or marked hyperglycemia (13). Letourneau and colleagues (2017), observed that 78.8% of the cohort with variants in the KATP channel showed DKA (39), as seen in our patient. This high prevalence may be explained due to the delay in the diagnosis. Besides, clinical manifestations in the central nervous system in patients with variants in the *KCNJ11* are not rare. Bowman and coworkers (2018) observed such features in more than 60% of patients with PNDM (14).

The patient presented in this study carries the c.754G>A;p.(Val252Met) variant in the *KCNJ11* gene. Despite suboptimal treatment adherence, a favorable response to SU was observed when the patient complied with the treatment, in contrast to insulin therapy.

Patients with variants in the KATP channel who were previously treated by insulin injections exhibited improvement when the treatment was switched to SU (37, 38). SU is an oral drug that facilitates treatment acceptance. SU also provides better glycemic control with a reduction in HbA1c by an average of 2.2% in the first year after the switch from insulin to SU, an increase in C-peptide plasma levels, and a reduction in the risk of complications associated with diabetes. Severe hypoglycemic episodes and side effects are not common, but patients in rare cases can experience gastrointestinal disturbance (e.g., diarrhea, nausea, and weight loss due to reduced appetite) (14).

The SU dose required has been related to the patient’s age at the time of treatment initiation. A higher dose is required as the age at which treatment is initiated increases. Besides, patients can require additional drugs when SU is initiated at older ages (23). Despite the late initiation of SU in our patient, at 15 years old, he shows a good response to a low dose (0.1 mg/kg/day) of SU with no other hypoglycemic medication required.

The early age at the time of initiation of SU also seems to be associated with an improvement in neurological and psychomotor development (e.g., hypotonia, muscle tone, muscle weakness, gross and fine motor skills, epilepsy, intelligence scores, visual attention deficits, learning difficulties, speech, and gesture conception and realization) (14, 30). However, in contrast with the restoration of normoglycemia, the recovery of the central nervous system (CNS) features is not common. Even though SU crosses the blood-brain barrier (BBB), acting in its receptors in the brain, it is rapidly removed not being sufficient to influence neuronal function (40).

One of the aspects that influences whether the switch to SU will be successful seems to be determined by the genetic variants. The successful switch in the patient reported here may be explained by the findings of Babiker and colleagues (2016), who performed an *in vitro* experiment to analyze the response of mutated KATP channels to tolbutamide, a type of SU. They compared these results with the response of patients who switched from insulin to SU. Tolbutamide (0.5 mmol/l) blocked more than 95% of KATP channels mutated for p.(Val252Met), and all three patients carrying this variant responded positively to SU (24). However, in cases with the SU transference after the first decade of diagnose, minor improvement in CNS features may be seen (19).

More than 70 residues in Kirk6.2 have been reported associated with a wide clinical spectrum of DM (15). For instance, variants that lie in residues that interact with ATP (ASN48, ARG50, ILE182, LYS185, TYR330, PHE333, and GLY334) or next to these residues, such as HIS46, impair ATP inhibition leading to NDM (15). The residue VAL252 lies close (3.8 Å) to HIS46, that is near HIS48 (4 Å) which in turn is predicted to interact with ATP, indicating that variants in the VAL252 residue, like p.(Val252Leu), can influence the ATP sensitivity (41).

The codon in position 252 seems to be an important cause for NDM when modified, since almost all possible changes (Methionine, glycine, alanine, and leucine), have been described as showing a wide phenotype described in the literature, except glutamic acid. The p.(Val252Met) variant was previously described by Fraser and coworkers (2012) in a literature survey, in two patients with PNDM and TNDM, but with no neurological involvement. In the same survey, they also described the variant p.(Val252Gly) in one patient with PNDM and iDEND syndrome, and p.(Val252Ala) in four patients (three with PNDM and one with TNDM), two of whom had no neurological abnormalities (42). This variant was described by Girard and coworkers (2006) in six patients with PNDM and one patient with TNDM carrying (41). Ješić and colleagues (2021) described the p.(Val252Leu) variant in a female infant diagnosed with hyperglycemia and glucosuria at 4 days of life. The genetic diagnostic and SU was started in the first month of life and after 10 years on SU, she maintained excellent glycemic control (the median of HbA1c was 5.9%) and has normal mental and social skills and glibenclamide dose was 0.3 mg/kg per day and does not show additional clinical features (22). Given that the same variant or variants within the same codon can lead to distinct phenotypes, we hypothesize that the age at SU initiation, together with additional modifying factors, influences the resulting phenotype. This is in line with previous findings suggesting that prolonged exposure to hyperglycemia may lead to a reduction in β-cell mass, consequently influencing treatment response and long-term phenotype (27).

Despite certain limitations of the present study, such as: the absence of genetic testing in family members; the patient’s poor adherence throughout the treatment; and the lack of neurocognitive assessments; This case illustrates the potential therapeutic advantages of initiating sulfonylurea treatment, even in individuals with a long-standing diagnosis of DEND syndrome. It also reinforces the critical role of genetic diagnosis, as early introduction of sulfonylureas may enhance metabolic regulation and, in certain cases, lead to neurological improvement.

## Data Availability

The datasets presented in this study can be found in online repositories. The names of the repository/repositories and accession number(s) can be found in the article/supplementary material.
